# Evaluation of factors that may influence the development of chronic kidney disease in patients with bipolar disorder treated with lithium.

**DOI:** 10.1192/j.eurpsy.2023.260

**Published:** 2023-07-19

**Authors:** N. Gutiérrez Mora, J. Torres Cortés, I. Esteban Avendaño, V. Burguera Vion, J. M. Montes Rodríguez

**Affiliations:** 1 Hospital Universitario Ramón y Cajal, Madrid, Spain; 2 Psychiatry; 3 Nephrology, Hospital Universitario Ramón y Cajal, Madrid, Spain

## Abstract

**Introduction:**

Bipolar disorder (BD) is a serious and chronic mental disease of mood. Lithium is used for treatment and studies have demonstrated that it is the most efficient drug, reducing suicide risk in a high percentage of patients. However, this drug has well known side effects, such as kidney damage. Lithium could cause chronic kidney disease, specially with the presence of other risk factors.

**Objectives:**

Observational and retrospective study of creatinine levels and glomerular filtration rates observed in blood analysis (follow-up period of 11 years). Sample size of 263 patients diagnosed of BD I and BD II in treatment with lithium. We used socio-demographic (age, sex) and clinic variables (diabetes mellitus, hypertension, use of nonsteroidal anti-inflammatory drugs (NSAIDs) and/or diuretics) to generate bivariate and multivariate analysis.

**Methods:**

Our main objective is to analyze the deterioration of kidney function and the development of chronic kidney disease that chronic treatment with lithium can induce in patients with BD. Our secondary objective is to determine variables which could promote the development of chronic kidney disease, and to assess if these variables could be considered as risk factors during the treatment with lithium.

**Results:**

11,3 % of patients in our study developed chronic kidney disease during monitoring. The deterioration of GFR in patients in treatment with lithium was significantly associated with female sex and NSAIDs consumption. A trend towards statistical significance was found regarding the use of diuretics (p=0,060). No statistical significance was found between diabetes mellitus, hypertension or type of BD and the deterioration of kidney function in our sample. An inverse association was found between the GFR decline and the age but no statistical significance was demonstrated.

**Conclusions:**

We conclude that female sex and use of NSAIDs are predicting factors of GFR decline in patients with BD in chronic treatment with lithium. We must take into account these drugs or even avoid concomitant treatment (lithium and NSAIDs) in order to prevent chronic kidney disease. In addition to it, we should recommend careful use of diuretics during treatment with lithium because of risk of dehydration. Diabetes mellitus and hypertension have universally been associated to increase risk of development of chronic kidney disease. However, we have not found statistical significance in our study. Therefore, research should be done in order to determine specific risk factors in this group of patients and, consequently, optimize their treatment.

**Disclosure of Interest:**

None Declared

